# Intervenciones de prevención sobre el consumo de alcohol en jóvenes universitarios*

**DOI:** 10.15649/cuidarte.2388

**Published:** 2022-10-20

**Authors:** Nathalia Rodríguez Sierra, Evelyn Sánchez Rodríguez, Ruth Alexandra Castiblanco Montañez, Ana Julia Carrillo Algarra, Sandra Milena Hernández-Zambrano

**Affiliations:** 1 . Universidad Pedagógica y Tecnológica de Colombia. Tunja, Colombia. Email: nathalia.rodriguez01@uptc.edu.co Universidad Pedagógica y Tecnológica de Colombia Universidad Pedagógica y Tecnológica de Colombia Tunja Colombia nathalia.rodriguez01@uptc.edu.co; 2 . Universidad Pedagógica y Tecnológica de Colombia. Tunja, Colombia. Email: evelyn.sanchez@uptc.edu.co Universidad Pedagógica y Tecnológica de Colombia Universidad Pedagógica y Tecnológica de Colombia Tunja Colombia evelyn.sanchez@uptc.edu.co; 3 . Fundación Universitaria de Ciencias de la Salud (FUCS): Bogotá, Colombia. Email: racastiblanco@fucsalud.edu.co Fundación Universitaria de Ciencias de la Salud Bogotá Colombia racastiblanco@fucsalud.edu.co; 4 . Fundación Universitaria de Ciencias de la Salud (FUCS): Bogotá, Colombia. Email: ajcarrillo@fucsalud.edu.co Fundación Universitaria de Ciencias de la Salud Bogotá Colombia ajcarrillo@fucsalud.edu.co; 5 . Fundación Universitaria de Ciencias de la Salud (FUCS): Bogotá, Colombia. Email: smhernandez3@fucsalud.edu.co Fundación Universitaria de Ciencias de la Salud Bogotá Colombia smhernandez3@fucsalud.edu.co

**Keywords:** Estudiantes Universitarios, Consumo de bebidas alcohólicas, Prevención Primaria, Educación en salud, University students, Alcohol Drinking, Primary Prevention, Health Education, Estudantes universitarios, Consumo de bebidas alcoólicas, Prevencao Primária, Educacao em saúde

## Abstract

**Introducción:**

La ingesta de alcohol está condicionada por aspectos individuales y culturales.

**Objetivo:**

Identificar el efecto de intervenciones realizadas en el contexto latinoamericano sobre pautas de consumo o factores de riesgo asociados al consumo de alcohol en jóvenes universitarios.

**Materiales y Métodos:**

Revisión sistemática a partir de la pregunta PICO, Se realizó búsqueda desde abril a agosto del 2020 en las bases de PubMed, CUIDEN, BVS, Scielo, Google Scholar y Repositorios Gubernamentales. Se utilizaron descriptores DeCS y MeSH, en español, inglés y portugués con los operadores AND y OR. Criterios de elegibilidad: estudios experimentales y cuasi experimentales publicados entre 2014 y 2020. Se obtuvieron 49 artículos, la lectura crítica permitió seleccionar 8 a los cuales se les aplicaron las escalas AMSTAR2, TREND y CONSORT quedando 6 artículos para análisis. Según la Resolución 008430/93, Artículo 10, se consideró como investigación sin riesgo.

**Resultados:**

Intervenciones unicomponente reportaron efectos sobre creencias conductuales, actitudes, conocimiento de la sustancia, rendimiento académico, menor frecuencia de consumo y no conducir bajo efectos del alcohol. Las intervenciones multicomponente disminuyen en 3.03% el riesgo de consumo y reportan percepción positiva respecto a la utilidad de las actividades desarrolladas, satisfacción de expectativas, satisfacción general, calidad de materiales empleados, asistencia y puntualidad.

**Discusión:**

Los estudios sugieren implementar nuevas intervenciones, estrategias y políticas en salud primaria para promover una transformación social, educativa y sanitaria qdue generen un impacto favorable para mitigar la problemática actual e consumo de alcohol en jóvenes universitarios.

**Conclusiones:**

Esta revisión sistemática permitió sintetizar y valorar la evidencia disponible frente a intervenciones unicomponente y multicomponente realizadas en el contexto latinoamericano y del caribe que modifican positivamente factores de riesgo y pautas de consumo en jóvenes universitarios.

## Abstract (en)

**Introduction:**

Individual and cultural aspects condition alcohol intake.

**Objective:**

To identify the effect of interventions conducted in Latin America on consumption patterns or associated risk factors for alcohol consumption among young university students.

**Materials and Methods:**

Systematic review based on a PICO question. A literature search was conducted from April to August 2020 in PubMed, CUIDEN, BVS, Scielo, Google Scholar, and Governmental Repositories. DeCS and MeSH descriptors in Spanish, English, and Portuguese were used with Boolean operators AND and OR. Eligibility criteria included experimental and quasi-experimental studies published between 2014 and 2020. Forty-nine articles were identified, and critical reading allowed the selection of 8 articles to which the AMSTAR2, TREND, and CONSORT appraisal tools were used, leaving 6 articles for analysis. In accordance with Article 10 of Resolution 008430/93, this was considered risk-free research.

**Results:**

Single-component interventions reported effects on behavioral beliefs, attitudes, knowledge of the substance, academic performance, lower frequency of drinking, and not driving under the influence of alcohol. Multicomponent interventions reduced consumption risk by 3.03% and reported positive perceptions regarding the usefulness of activities, expectations fulfillment, overall satisfaction, quality of materials used, attendance, and punctuality.

**Discussion:**

The studies suggest implementing new interventions, strategies and policies in primary health to promote a social, educational and health transformation that generate a favorable impact to mitigate the current problem of alcohol consumption in university students.

**Conclusions:**

This systematic review made it possible to synthesize and evaluate the available evidence on single- and multicomponent interventions in Latin America and the Caribbean that positively modify risk factors and consumption patterns in young university students.

## Resumo

**Introdujo:**

A ingestao de álcool é condicionada por aspectos individuáis e culturáis.

**Objetivo:**

Identificar o efeito de intervencóes realizadas no contexto latino-americano sobre os padróes de consumo ou fatores de risco associados ao consumo de álcool em estudantes universitários.

**Materiais e Métodos:**

Revisao sistemática com base na pergunta PICO, realizada de abril a agosto de 2020 nas bases de dados PubMed, CUIDEN, BVS, Scielo, Google Scholar e Government Repositories. Foram utilizados os descritores DeCS e MeSH, em espanhol, ingles e portugués com operadores AND e OR. Critérios de elegibilidade: estudos experimentais e quase-experimentais publicados entre 2014 e 2020. Obtiveram-se 49 artigos, a leitura crítica permitiu selecionar 8, aos quais foram aplicadas as escalas AMSTAR2, TREND e CONSORT, restando 6 artigos para análise. De acordo com a Resolucao 008430/93, artigo 10, foi considerada pesquisa livre de risco.

**Resultados:**

Intervencóes unicomponentes relataram efeitos sobre crencas comportamentais, atitudes, conhecimento da substancia, desempenho académico, menor frequéncia de consumo e nao dirigir sob efeito de álcool. As intervencóes multicomponentes reduzem o risco de consumo em 3,03% e relatam uma percepcao positiva quanto a utilidade das atividades realizadas, satisfago das expectativas, satisfago geral, qualidade dos materiais utilizados, assiduidade e pontualidade.

**Discussáo:**

Os estudos sugerem a implementacao de novas intervencóes, estratégias e políticas na atencao primária a saúde para promover uma transformacao social, educacional e sanitária que gere impacto favorável para mitigar o problema atual do consumo de álcool em estudantes universitários

**Conclusóes:**

Esta revisao sistemática permitiu sintetizar e avaliar as evidencias disponíveis sobre intervencóes unicomponentes e multicomponentes realizadas no contexto latino-americano e caribenho que modificam positivamente os fatores de risco e os padróes de consumo em estudantes universitários.

## Introducción

Según la Organización Panamericana de la Salud (OPS), la prevalencia de episodios de consumo excesivo de alcohol en jóvenes se relaciona con diferentes factores que determinan comportamientos como, el inicio de consumo a temprana edad y la ingesta desmesurada y poco controlada, condicionadas por la cultura y las normas, fenómeno que se refleja en Latinoamérica y el Caribe donde celebran fiestas y ocasiones especiales con consumo de alcohol, además los horarios de venta restringidos y la reglamentación no logran controlar su uso^1^.

Respecto a los Objetivos de Desarrollo Sostenible (ODS), el consumo de alcohol afecta 14 de los 17 objetivos, y genera múltiples daños de salud, sociales y económicos^1^. Dada su importancia es una prioridad política, social, de salud, y los países han aumentado sus esfuerzos por formular y actualizar políticas, planes y programas, aunque no se han establecido indicadores para medir la reducción del consumo nocivo de alcohol en la estrategia mundial de la Organización Mundial de la Salud (OMS) o en el plan de acción regional. Además, las políticas no son coste eficaces para reducir la ingesta de alcohol, por ello la OMS y los ODS se han propuesto reducir el consumo, sin embargo la OPS^2^ reporta aumento en la prevalencia de episodios de ingesta, con tendencia al incremento especialmente en la población más joven asociada a diferentes factores como la disponibilidad de bebidas alcohólicas y su fácil adquisición generando un impacto negativo en el desarrollo económico de la sociedad, que interfiere el objetivo de una reducción relativa del 10% para el 2025^1^.

A nivel mundial, 155 millones de jóvenes consumen alcohol, y el continente americano es el segundo en magnitud, esta práctica impacta de manera diferente en mujeres y hombres jóvenes, porque los hombres presentan más tolerancia al alcohol por ello, requieren mayores dosis que las mujeres^1^. En Chile, el consumo excesivo de alcohol es un problema de salud, anualmente los hombres ingieren 13,9 litros y las mujeres 5,5 litros,^3^ con estos datos se infiere que la situación es alarmante, en América Latina y el Caribe. En Colombia según el Observatorio de Drogas y el Ministerio de Educación Nacional en 2016 en cuanto al consumo de alcohol juvenil, el 68,1% de los hombres y el 70,4% de mujeres declararon haber consumido alguna bebida alcohólica en su vida; siendo los jóvenes universitarios quienes registran la cifra más alta con un 95,8%^4^. Respecto a el reporte de otros países latinoamericanos en 2012 donde indicó que en Ecuador 88,7%, Perú 87,5%, Bolivia 77,1%, España (58%) y Chile (64,2%), cifras que muestran una condición de consumo perjudicial que representa un riesgo psicosocial, con un rango de edad de mayor consumo de licor entre los 17 y 25 años^5^.

Esta práctica es un factor de riesgo transversal que afecta muchas áreas de la Agenda 2030, porque genera enfermedades crónicas, y problemas psicosociales como violencia, abandono, maltrato y ausentismo en el lugar de trabajo y estudio, entre otros^6^^,^^7^. Además, afecta el ámbito familiar, social, económico, político y se origina en las normas sociales que favorecen el consumo de alcohol^8^^,^^9^, el fácil acceso, la publicidad poco controlada que llega a cualquier tipo de público, aspectos que influyen para que se de esta práctica en jóvenes de manera temprana y sea socialmente aceptado y normalizado^10^^,^^11^.

Respecto a estudios sobre consumo de alcohol en jóvenes universitarios en el contexto Latinoamericano y del Caribe, se identificaron investigaciones de diseño observacional descriptivo, realizadas en países como México, Ecuador y Colombia que reportan un alto índice de ingesta de alcohol, incremento de consumo de riesgo y perjudicial especialmente en los hombres, sumado al riesgo de alcoholismo y dependencia^10^^-^^15^. Sin embargo, son escasos los estudios de síntesis de evidencias que analizan el efecto de intervenciones para mitigar o prevenir el consumo de alcohol en jóvenes universitarios, ya que la mayor parte se han realizado con población anglosajona^16^ en general, no siendo específicos en jóvenes universitarios, factor que supone una brecha cultural por las diferencias en los hábitos, representaciones sociales, prácticas y estructuras institucionales particulares de los contextos, que son determinantes en el efecto de las intervenciones^6,8-(^^17^. Por ello, en materia de políticas y programas de atención, se justifica la identificación y análisis de evidencias sobre este fenómeno en este contexto específico, dados los beneficios en la construcción de políticas públicas, planes y programas de sustancias psicoactivas y en la toma de decisiones en el ámbito clínico y comunitario. Por lo anterior, el objetivo de este estudio fue identificar el efecto de las intervenciones realizadas en el contexto Latinoamericano y del Caribe sobre pautas de consumo o factores de riesgo asociados al consumo de alcohol en los jóvenes universitarios.

## Materiales y Métodos

Revisión sistemática a partir de la pregunta PICO, ¿Cuál es el efecto de las intervenciones realizadas en el contexto Latinoamericano y del Caribe sobre pautas o factores de riesgo asociados al consumo de alcohol en los jóvenes universitarios?, mediante un riguroso proceso de búsqueda que permita sintetizar la evidencia para analizar el efecto de las intervenciones reportadas.

Se definieron como criterios de elegibilidad, estudios experimentales y cuasiexperimentales, de intervenciones unicomponente y multicomponente orientadas a prevenir o mitigar el consumo, publicados del 2014 al 2020, en español, inglés y portugués que incluyeran jóvenes universitarios Latinoamericanos y del Caribe, de ambos sexos, entre los 18 y 24 años.

La ecuación de búsqueda fue construida en tres idiomas Español, Inglés y Portugués utilizando descriptores MeSH y los operadores booleanos AND y OR "University Students"[Mesh]) "Alcohol Drinking"[Mesh] "Young Adult"[Mesh]) "Leisure Activities"[Majr] “Adolescent"[Mesh], Alcohol Drinking in College"[Mesh]) "Young Adult"[Mesh]. Se rastreó la información a través de metabuscadores, interfaz, repositorios, bases de datos y banco de artículos como: PubMed, CUIDEN, BVS, Scielo, Google Scholar y Repositorios Gubernamentales, se accedió a través de catálogos de la Universidad Pedagógica y Tecnológica de Colombia y la Fundación Universitaria de Ciencias de la Salud, la búsqueda se realizó en el primer semestre de 2020.

**Figura 1:**
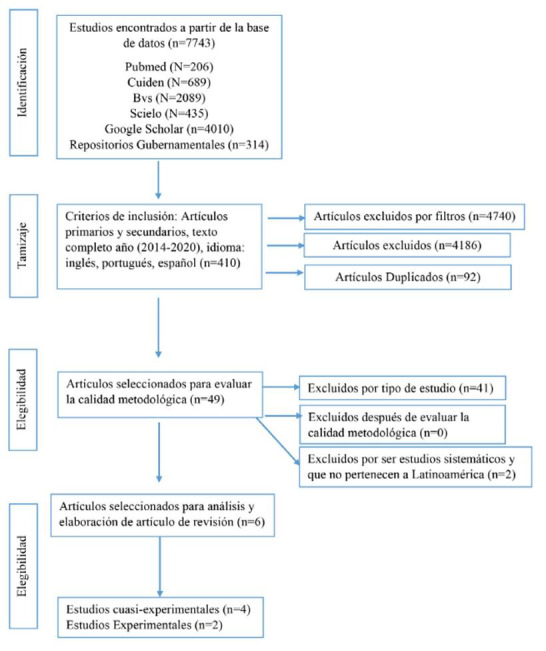
Diagrama prisma de la búsqueda sistemática

La selección de artículos se realizó por títulos, posteriormente por resúmenes y textos completos, finalmente las variables: Objetivo, metodología, población, intervención, principales resultados, efectos de las intervenciones y conclusiones se registraron en una matriz de Excel^18^. Para la lectura crítica se utilizaron los instrumentos AMSTAR-2^19^ para revisiones sistemáticas, para estudios experimentales, CONSORT^20^ y TREND,^21^ para estudios cuasi experimentales, la información del proceso de búsqueda se registró en el diagrama de flujo de la Figura 1.

Posterior a esto se utilizó la propuesta del Instituto Joanna Briggs^22^ para asignación de grado de evidencia; asimismo, se extrajeron los resultados de las intervenciones y las variables de resultado para cada estudio de manera que se pudiera dar cuenta de su efecto en la prevención del consumo en la población de enfoque. De acuerdo con la Resolución 008430/93 se consideró como investigación sin riesgo ^23^.

## Resultados

La búsqueda inicial reportó 7743 artículos. Como se ilustra en la Figura 1 después de utilizar filtros se analizaron 49 artículos, seleccionados por lectura crítica y verificación por escalas 6 estudios para el análisis de datos, 4 estudios cuasi-experimentales y 2 estudios experimentales, realizados en: Brasil, Colombia, Ecuador, México y Cuba, publicados en español y portugués. El año con mayor número de publicaciones fue el 2014, los participantes fueron jóvenes estudiantes universitarios. En la Tabla 1 se describen las principales características de la muestra bibliográfica.

Respecto al diseño y objetivos de los estudios, dos de ellos se enfocan en comprobar la eficacia de programas preventivos, en diseñar, implementar y evaluar programas de prevención frente al consumo de alcohol en jóvenes universitarios, los otros dos estudios cuasiexperimentales se enfocaron en implementar una intervención educativa y la formación de líderes para prevenir la drogodependencia en jóvenes universitarios; un estudio experimental se enfocó en estimular la no conducción bajo los efectos del alcohol y las estrategias preventivas en jóvenes, mientras el otro, abordó el problema mediante el uso de Audit e Intervención Breve (IB) para cambiar el patrón y reducir el consumo de alcohol.

En cuanto a la población de estudio las investigaciones se realizaron en jóvenes, dos de los estudios utilizó la aleatorización para la selección o asignación de los sujetos de estudio, el resto de los estudios no informan el tipo de selección.

**Tabla 1:** Características de la muestra bibliográfica

Referencia bibliográfica	Idioma/ país	Objetivo	Diseño metodológico/Nivel de evidencia	Participantes
Albán Obando J. J. (2017) ^24^	Español/Ecuador	Comprobar la eficacia del programa preventivo y motivacional en el incremento del rendimiento académico, el alto nivel de motivación por los estudios y la disminución del consumo de alcohol en jóvenes universitarios de la Escuela de Psicología de la Universidad Técnica de Babahoyo.	Estudio cuasi- experimental. Nivel de evidencia: 2d	Grupo control (30 estudiantes) y un grupo experimental (30 estudiantes), de sexo masculino y femenino, entre los 19 y 24 años, condición socio económica baja y media, que comparten la pertenencia a un grupo de riesgo con tendencia al uso de alcohol y presentan poca motivación por los estudios.
Almeida ND, Roazzi A. (2014)^25^	Portugués/Brasil	Estimular la no conducción bajo efectos del alcohol y las estrategias preventivas, así como aportar nuevos modelos (medios de comunicación, por ejemplo) para dilucidar con mayor realismo la complejidad de este comportamiento en esta población.	Estudio experimental. Nivel de evidencia: 1c	163 estudiantes universitarios, 60.7% mujeres y 39.3% mujeres. Varón, entre 18 y 42 años. Distribuidos en Grupo Experimental 1-GE1= 42 participantes, el Grupo Experimental 2-GE2 = 41 participantes, el control- placebo-GCP=40 participantes y el solo control-GSC = 40 participantes. La muestra fue por conveniencia
Cabrera Rodríguez D, Ricardo Díaz et al. (2016)^26^	Español/Cuba	Implementar una intervención educativa para prevenir la drogodependencia en estudiantes de medicina del Municipio de Cacocum.	Estudio cuasi experimental. Nivel de evidencia:2c	42 estudiantes de sexto año de medicina, seleccionados por un muestreo no probabilístico intencionado.
Salazar Mendoza J. (2014) ^27^	Español/México	Formar líderes en la promoción y prevención contra las adicciones y multiplicadores de la capacitación que reciben, para fortalecer prevención de adicciones en la comunidad universitaria y en general, además formar estudiantes de enfermería conscientes de la necesidad de atención preventiva, diagnóstica, de tratamiento y rehabilitación en la atención de personas con adicciones y población en riesgo.	Estudio cuasi experimental. Nivel de evidencia: 2d	Grupo experimental (10 estudiantes) y de control (10 estudiantes)
Sawicki, Wanda Cristina, et al (2018) ^28^	Portugués/Brasil	Investigar el consumo de alcohol en estudiantes de enfermería y evaluar la intervención. Principalmente para los abusadores del alcohol.	Estudio experimental. Nivel de evidencia: 2c	Participaron 281 jóvenes universitarios
Muñoz Ortega, María Liliana. (2014) ^29^	Español/Colombia	Diseñar, implementar y evaluar un programa de prevención al consumo de alcohol en jóvenes universitarios en la ciudad de Bogotá.	Estudio cuasi experimental. Nivel de evidencia: 2c	Participaron 101 estudiantes universitarios (hombres y mujeres) de las diferentes carreras y jornadas diurna y nocturna, entre 16 y 31 años, quienes en siete grupos focales dieron sus ideas. Finalmente, se organizó un grupo de ocho estudiantes, los cuales, bajo la denominación de líderes, analizaron la información de los grupos e hicieron la propuesta del programa.

Respecto a las intervenciones breves y psicoeducativas, los programas preventivos motivacionales, la construcción de comunicaciones persuasivas y acciones educativas, técnicas participativas, reportadas en los estudios para prevenir o minimizar el consumo de alcohol, se destaca que cuatro de los estudios presentaron intervenciones unicomponente orientadas al desarrollo de programas psicoeducativos o intervención breve; mientras que los otros dos estudios presentan una intervención multicomponente, basadas en sesiones-acciones educativas, técnicas participativas (presentación, motivación, participación y de cierre) de los jóvenes universitarios.

En el caso de los estudios unicomponente se reportaron los siguientes efectos de las intervenciones en variables como consumo del alcohol, creencias conductuales, actitudes y conocimientos respecto a la sustancia, obtuvo un resultado de (p= <0,001), y reporta que después de haber aplicado los talleres de prevención y motivación un 50% de los estudiantes estuvieron más interesados en aprender cosas nuevas de la carrera, así mismo evidenció un aumento del nivel de rendimiento muy bueno 50 % y el 20% tiene un nivel de rendimiento excelente^24^^),^ en el caso de las variables: creencias conductuales generales y la actitud, adoptar comportamiento de no ingerir alcohol y el direccionamiento, el efecto de las intervenciones que fueron construcción de comunicaciones persuasivas, p= < 0,016, muestra un efecto positivo en el grupo experimental expuesto a comunicación persuasiva positiva, en relación con el grupo control que recibió comunicación irrelevante, con respecto a la variable dependiente, la intención conductual de no consumir bebidas alcohólicas y conducir en jóvenes universitarios.

Los resultados del estudio de Almeida y Roazzi^25^ respecto a las diferentes variables fueron: Intervención de Enfermería (p= <0.05) demostró que las medias de actitudes se incrementaron en el grupo experimental después de la intervención, sin embargo, para el grupo control disminuyó y no se mantuvo en el rango inicial. El nivel de conocimientos sobre los efectos del consumo de alcohol fue mayor en el grupo experimental que el control, antes de la intervención, (p= <0.039), indicando que el 100% de la población tiene un consumo de alcohol previo, diferencia que no fue estadísticamente significativa, después de la intervención el 100% de los integrantes del grupo experimental disminuyó significativamente el consumo^24^.

En lo que corresponde a las variables: menor frecuencia consumo de alcohol (<5 dosis) (p= <0,0086) indica una disminución en la frecuencia de consumo de 5 dosis o más por episodio, comenzando a no consumir o consumir menos de una vez al mes, después de la aplicación de la intervención en comparación con las etapas anteriores del estudio, en las variable No intervenidos >dosis (p= <0.0335) y No intervenidos >frecuencia (p= <0.0114) se demostró que el consumo fue superior en los No intervenidos que en los intervenidos, en la variable de Renta familiar hasta 7 MW (p= <0,0427) comenzaron a consumir bebidas alcohólicas con menor frecuencia a partir de la aplicación de la intervención lo cual concuerda con la variable Menor edad de los jóvenes (p= <0.0001) ya que es menor la frecuencia de ingesta de alcohol tras la aplicación de la misma 2° IB^25^.

De acuerdo con las intervenciones multicomponente las variables encontradas indican que los jóvenes que están en riesgo mínimo (79,63%), pero diversos factores interfirieron en la implementación, y los resultados no mostraron diferencias estadísticamente significativas entre las evaluaciones antes y después del programa, sin embargo el estudio de Muñoz.^29^ reporta que los efectos fueron positivos dado que la diferencia se encuentra en un pequeño porcentaje, el mayor porcentaje de jóvenes universitarios en ambas evaluaciones se ubicó en un riesgo mínimo, con 2,23% superior en la evaluación final. En el riesgo de consumo se ve una disminución de 3,03 % de jóvenes en la evaluación final. Cabrera et al.^26^ reportó utilidad de las actividades desarrolladas 100%, satisfacción de expectativas 90,47%, satisfacción general 85,71%, calidad de los materiales empleados 80,95%, asistencia y puntualidad 71,43%, observando que la estrategia educativa implementada se pudo valorar como satisfactoria y alcanzó la excelencia en la mayoría de las variables que se exploraron, ningún aspecto fue evaluado como malo ni regular, acción que fue favorable pues se lograron las expectativas que los estudiantes tenían con el programa. La anterior información se encuentra consignada en la Tabla 2.

**Tabla 2:**
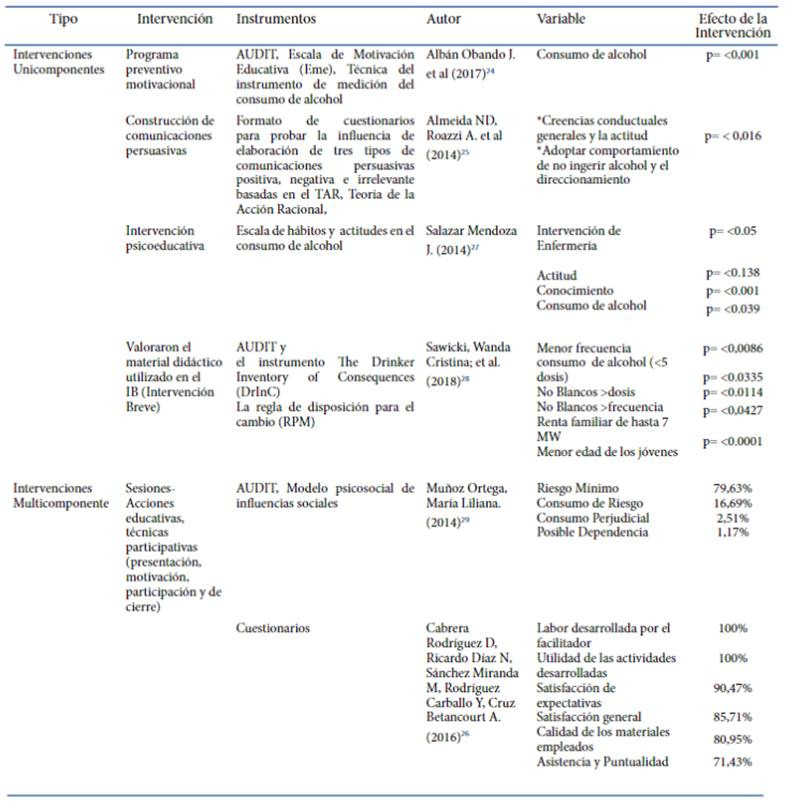
Costos totales del programa “Cuidando a los cuidadores”

## Discusión

Dentro de los hallazgos de esta revisión sistemática uno de los resultados principales es que la intervención breve demuestra efecto en la reducción de la frecuencia del consumo de alcohol (<5 dosis) e indica que después de haber aplicado los talleres de prevención y motivación un 50% de los estudiantes estuvieron más interesados en aprender cosas nuevas de la carrera y mejoran el rendimiento académico^24^. Con respecto a otras intervenciones psicoeducativas el 100% de la población consumidora de alcohol después de la intervención disminuyó significativamente el consumo^25^.

En el caso de los estudios de tipo multicomponentes, en el primer estudio se evaluaron por porcentajes las intervenciones, se usaron sesiones educativas, implementado la encuesta AUDIT antes de las sesiones^29^, el segundo estudio también utilizó sesiones educativas e hizo mediciones antes y después, evidenciando que el impacto de las sesiones fue favorable en la variables de la labor desarrollada por el facilitador y la utilidad de las mismas, el resultado generado fue que la población participante tuvo mayor conciencia frente a la importancia de la disminución del consumo de alcohol y las herramientas de prevención enfocadas en modificar los hábitos y los conocimientos respecto a la ingesta de alcohol^26^, este estudio según grado de recomendación por su eficacia puede ser usado y tenido en cuenta como base para la formulación de otros estudios sobre este fenómeno.

Con respecto al efecto de las intervenciones breves, se logró verificar una disminución significativa en el patrón de consumo de alcohol nocivo y dependiente para consumidores de bajo riesgo; así mismo, se identificó una diferencia significativa en el número de dosis consumida y una disminución en la frecuencia de ingesta de alcohol tras la aplicación de la segunda intervención breve^28^; un estudio publicado en el contexto anglosajón sugiere que las intervenciones breves son factibles y aceptables en entornos comunitarios de trabajo con jóvenes, sin embargo, lo que fue más importante de estas intervenciones es que se perciben no como una intervención discreta o formal como tal, sino más como una forma de trabajar con los jóvenes en torno al consumo colectivo de alcohol^15^^), (^^30^. En una revisión de ensayos controlados compararon la intervención breve con una intervención mínima o con ninguna logrando identificar que durante un año de seguimiento las personas que recibieron la intervención breve bebieron menos que los participantes del grupo control^31^.

Esta evidencia soporta que la implementación de las intervenciones breves logra mitigar o generar un impacto positivo en la reducción del consumo de alcohol. También lo encontrado en la literatura evidencia que gracias a los avances tecnológicos se han implementado intervenciones breves por computadora y por teléfono móvil para reducir el consumo de alcohol y sustancias psicoactivas en los jóvenes ya que son atractivas porque permiten a los usuarios controlar el ritmo de la intervención, garantizar la privacidad y tener un alcance más amplio a bajo costo^32^.

De acuerdo a los programas psicoeducativos que implementan sesiones enmarcadas en el Modelo de Promoción de la Salud, que están diseñadas para obtener resultados sobre las actitudes, conocimiento y consumo de la adopción de herramientas indispensables de resiliencia; los resultados obtenidos fueron satisfactorios al término de la intervención, según el análisis de las comparaciones antes y después de la intervención, para la disminución del consumo de alcohol^27^. Es así como otros artículos que trabajan intervenciones similares suelen concentrarse en la formación de habilidades sociales con el objetivo de capacitar a los jóvenes, potenciando la participación comunitaria a través de actividades de educación para la salud^33^^-^^36^.

En el caso del estudio con intervenciones psicoeducativas, el análisis se realizó en base a la propuesta de programas de prevención con un enfoque participativo, al comparar los datos de las evaluaciones inicial y final, se encontró que las distribuciones se presentaron de manera no significativa, por el corto tiempo, por tanto, sugieren planear intervenciones de mayor tiempo que permitan a los estudiantes una apropiación de estrategias en el consumo de alcohol, además de implementar el desarrollo de habilidades, toma de decisiones de los adolescentes, la búsqueda de identidad personal, el establecimiento de metas o la práctica y el refuerzo de habilidades y conductas^29^^,^^37^^-^^39^.

Existe evidencia de que los jóvenes son más vulnerables a los daños relacionados con el consumo de alcohol^40^ que las personas mayores^41^, uno los resultados del presente estudio generan la posibilidad de que las intervenciones puntuales o breves (una sola sesión) podrían no ser suficientes para generar efecto a largo plazo en la prevención de consumo de alcohol. Este hallazgo sería consistente con los resultados de distintos artículos, en los que se pone de manifiesto que es necesario mejorar el acceso a las estrategias presenciales, seguimiento y tratamiento para la reducción de problemas relacionados con el consumo de alcohol^42^; aunque reportan que las personas que reciben intervención breve beben menos que los que no reciben ninguna intervención^32^^,^^43^, se observa que intervenciones como (mhealth) acceden a plataformas móviles y son atractivas porque permiten a los usuarios controlar el ritmo de la intervención, garantizar la privacidad, flexibilidad, oportunidad y tener un alcance más amplio a un bajo costo, revolucionan la capacidad de monitorear el comportamiento de las personas y favorecen a los jóvenes en regiones con difícil acceso a información sobre el consumo de alcohol^32^^,^^41^^,^^44^ sin embargo, dentro de la evaluación del uso de estrategias de captación, no han demostrado ser efectivas en gran parte de la población^38^^,^^45^.

Al respecto en término de implicaciones para la práctica, se sugiere que se implementen nuevas intervenciones, estrategias y políticas en salud primaria que plantean una transformación social, educativa y sanitaria, generan un impacto favorable para mitigar la problemática actual de consumo de alcohol en los jóvenes especialmente en Latinoamérica, y promueven hábitos, estilos y conductas de vida saludables entre los jóvenes para aumentar los factores protectores y disminuir factores de riesgo^46^^-^^48^. como acciones que puede desarrollar enfermería, dada su facilidad para contactarse con las comunidades e identificar patrones de comportamiento y de consumo de alcohol que pueden poner en riesgo a los jóvenes^49^^,^^50^^,^ dado que es un fenómeno que afecta seriamente la salud de este grupo poblacional e implica un alto costo social^51^^,^^52^, por lo que representa una prioridad de acción para los profesionales en salud. La evidencia presentada puede ser de utilidad para investigadores, personal de salud y personal en formación que estén interesados en indagar y generar nuevas intervenciones, estrategias y producción de documentos encaminados a minimizar el consumo de alcohol en esta población.

La presente revisión tiene algunas limitaciones el no incluir meta análisis y por tanto no se controlaron los posibles sesgos de selección.

## Conclusión

Esta revisión sistemática permitió sintetizar y valorar la evidencia disponible frente a diferentes intervenciones realizadas en el contexto latinoamericano y del caribe con el fin de modificar pautas de consumo o factores de riesgo asociados al consumo de alcohol en los jóvenes universitarios. Se reportan los efectos de las intervenciones breves y psicoeducativas que tuvieron un efecto favorable, en la reducción del consumo de alcohol, se evidencio una disminución del 50% de los factores de riesgo y un aumento significativo de los factores protectores en comparación con el antes de la implantación de los programas.

Analizar el panorama general de las intervenciones a nivel Latinoamérica y el caribe permite evidenciar la evolución del abordaje a este fenómeno de interés para la ciencia ya que son muy escasos los estudios que se han encontrado que midan efecto de intervenciones en el problema del consumo de alcohol entre los jóvenes desde la perspectiva de los significados y las representaciones sociales. En la actualidad la prevalencia del consumo de alcohol a nivel mundial es preocupante por el uso y abuso generalizado de una gran parte de los jóvenes universitarios, que lo han adoptado como parte de sus actividades de recreación, ocio y de aceptación social por lo que se proponen estrategias y políticas públicas encaminadas a las intervenciones y programas que reduzcan o modifiquen las pautas de consumo de alcohol.
